# Repurposing the anticancer drug cisplatin with the aim of developing novel *Pseudomonas aeruginosa* infection control agents

**DOI:** 10.3762/bjoc.14.284

**Published:** 2018-12-14

**Authors:** Mingjun Yuan, Song Lin Chua, Yang Liu, Daniela I Drautz-Moses, Joey Kuok Hoong Yam, Thet Tun Aung, Roger W Beuerman, May Margarette Santillan Salido, Stephan C Schuster, Choon-Hong Tan, Michael Givskov, Liang Yang, Thomas E Nielsen

**Affiliations:** 1 Singapore Centre for Environmental Life Sciences Engineering (SCELSE), Nanyang Technological University, Singapore 637551; 2 Lee Kong Chian School of Medicine, Nanyang Technological University, Singapore 639798; 3 School of Biological Sciences, Nanyang Technological University, Singapore 639798; 4 Singapore Eye Research Institute, Singapore 169879; 5 SRP Neuroscience and Behavioural Disorders and Emerging Infectious Diseases, Duke-NUS, Singapore 169857; 6 Ophthalmology, Yong Loo Lin School of Medicine, National University of Singapore, Singapore 168751; 7 Division of Chemistry & Biological Chemistry, School of Physical & Mathematical Sciences, Nanyang Technological University, Singapore 637371; 8 Costerton Biofilm Center, Department of Immunology and Microbiology, University of Copenhagen, 2200 København N, Denmark

**Keywords:** biofilm, cisplatin, *Pseudomonas aeruginosa*, resistance, type III secretion

## Abstract

Antibiotic resistance threatens effective treatment of microbial infections globally. This situation has spurred the hunt for new antimicrobial compounds in both academia and the pharmaceutical industry. Here, we report how the widely used antitumor drug cisplatin may be repurposed as an effective antimicrobial against the nosocomial pathogen *Pseudomonas aeruginosa*. Cisplatin was found to effectively kill strains of *P. aeruginosa*. In such experiments, transcriptomic profiling showed upregulation of the *recA* gene, which is known to be important for DNA repair, implicating that cisplatin could interfere with DNA replication in *P. aeruginosa*. Cisplatin treatment significantly repressed the type III secretion system (T3SS), which is important for the secretion of exotoxins. Furthermore, cisplatin was also demonstrated to eradicate in vitro biofilms and in vivo biofilms in a murine keratitis model. This showed that cisplatin could be effectively used to eradicate biofilm infections which were otherwise difficult to be treated by conventional antibiotics. Although cisplatin is highly toxic for humans upon systemic exposure, a low toxicity was demonstrated with topical treatment. This indicated that higher-than-minimal inhibitory concentration (MIC) doses of cisplatin could be topically applied to treat persistent and recalcitrant *P. aeruginosa* infections.

## Introduction

*Pseudomonas aeruginosa* is a leading nosocomial pathogen which causes, among others, corneal, chronic otitis media, urinary tract (UTI) and respiratory tract infections [1]. *P. aeruginosa* is also the main cause of fatal infections in patients with cystic fibrosis (CF) [2] and cancer patients [3–4]. The success of *P. aeruginosa* as a leading pathogen is attributed to its ability to form resilient biofilms, resist antimicrobials and secrete virulence products.

Microbial cells resident in biofilms are encased by an extracellular matrix, which protects them from antimicrobial treatment and the host’s immune clearance [5]. Clinical *P. aeruginosa* isolates are mostly multidrug-resistant (MDR) strains [6], with robust ability to form biofilms [7]. *P. aeruginosa* also secretes virulence factors targeting important components of the immune system, such as the type III secretion systems (T3SS), which was shown to be associated with poor clinic outcomes in patients with lower respiratory infections [8] and ventilator-associated pneumonia [9]. Identifying antimicrobial compounds which actively target bacteria in the biofilm mode including virulence mechanisms that cripple immune defenses, may offer novel antimicrobial therapies against a variety of otherwise persistent *P. aeruginosa* infections.

Here, we screened our in-house collection of FDA-approved drugs and found that cisplatin was the most potent among several other Pt(II)-based compounds to kill *P. aeruginosa*. It was previously reported that cisplatin had antimicrobial effects on nosocomial pathogens, such as *Escherichia coli*, *Klebsiella pneumonia* and *Staphylococcus aureus* [10] and persister cells [11]. Transcriptomic analysis was employed to reveal the molecular mechanisms on how cisplatin inhibits the growth and production of virulence factors of *P. aeruginosa*. We also examined the effects of cisplatin treatment on in vitro *P. aeruginosa* biofilms and in a mouse model of corneal infection (keratitis). We showed that cisplatin is more effective than the clinically used antibiotic tobramycin in eradicating biofilms. Although cisplatin is highly toxic for intravenous applications, we showed that it has low toxicity when applied topically to wounds, as 25 mM (0.75 mg mL^−1^) did not have adverse effect on wound healing. This meant that higher doses (5–10 × MIC) of cisplatin could be safely used for topical applications. Given the low topical toxicity of cisplatin, it may be utilized as an attractive therapeutic agent for prevention and treatment of *P. aeruginosa* biofilm infections.

## Materials and Methods

### Bacterial strains and culture media

The bacterial strains used in this study are listed in Supporting Information File 1, Table S3. Luria–Bertani (LB) medium was used to maintain the bacterial strains. Growth assay and static biofilm cultivation were carried out at 37 °C in ABTGC (ABT minimal medium [12] supplemented with 0.4 g/L glucose and 0.4 g/L casamino acids). For marker selection in *P. aeruginosa*, 30 μg mL^−1^ gentamycin (Gm), 50 μg mL^−1^ tetracycline (Tc), 100 μg mL^−1^ streptomycin (Strep) or 200 μg mL^−1^ carbenicillin (Cb) were used, as appropriate.

### Platinum complexes and solution preparation for MIC assay

Cisplatin, transplatin, cDPCP, oxaliplatin, K_2_PtCl_4_ and *cis*-PtCl_2_(CH_3_CN)_2_ were purchased from Sigma-Aldrich were used as received. Platinum complexes *cis*-PtCl_2_(Py)_2_ [13–14] and *cis*-PtCl_2_(PPh_3_)_2_ [15] were prepared according to the reported procedure. For minimal inhibitory concentration (MIC) assays, cisplatin, cDPCP, oxaliplatin, K_2_PtCl_4_ and *cis*-PtCl_2_(CH_3_CN)_2_ were dissolved in saline solution (0.85% w/v) at 2.5 mM concentration, while transplatin, *cis*-PtCl_2_(Py)_2_ and *cis*-PtCl_2_(PPh_3_)_2_ were dissolved in DMF (v/v) at 2.5 mM concentration.

### Determination of minimal inhibitory concentration (MIC)

The MIC assays were performed using a microtiter broth dilution method as previously described (≈1 × 10^5^ cells) in the NACLAR guidelines [16]. Overnight cultures of bacterial strains were diluted in ABTGC medium. Cisplatin and other Pt-containing compounds were diluted from a stock solution with ABTGC medium at a concentration 10 times higher than the required range. 10 μL of each diluted solution of Pt compounds were added to each corresponding well of a 96-well microtiter plate (polypropylene, Costar) and 90 μL of diluted bacterial culture in ABTGC medium were added before serial dilutions. The plate was incubated at 37 °C for 16–18 h. MIC was taken as the lowest concentration where no visual growth (based on OD600) of bacteria was detected. The experiments were performed in triplicate and representative results were shown.

### RNA preparation

Bacterial cells were collected using the method described previously [17] with some modifications. Generally, PAO1 cells were cultivated either with (1.5 μM) or without cisplatin. The cells were harvested at the early-stationary phase (after approximately 8 h cultivation). Total RNA was extracted with an RNeasy Protect Bacteria Mini Kit with on-column DNase digestion (Qiagen). A Turbo DNA-free vigorous protocol was used for a second round of DNase treatment (Ambion). The 16S, 23S and 5S rRNA was removed using the Ribo-Zero Magnetic Kit (Bacteria) (Epicentre).

### RNA sequencing and data analysis

Gene expression analysis was conducted via Illumina RNA sequencing (RNA-Seq technology). RNA-Seq was conducted for two biological replicates of each sample. The rRNA-depleted RNA was fragmented to 150–200 bp fragments, then first and second strand cDNA were synthesized with a cDNA-synthesis kit (ThermoScientific), followed by end repair and adapter ligation. After 12 cycles of PCR enrichment, the quality of the libraries was assessed using the Bioanalyzer (Agilent Technologies, USA). The libraries were sequenced using the Illumina HiSeq 2500 platform with a paired-end protocol and read lengths of 100 nt.

The sequencing data was analyzed as described previously [12]. Sequence reads were mapped onto PAO1 reference genome using the CLC genomics Workbench 8.0 (CLC Bio-Qiagen, Aarhus, Denmark). The differentially expressed genes were identified by performing a negative binomial test using the DESeq [18] package of R/Bioconductor [19], using the cut off of fold-change larger than 2 and a BH (Benjamini-Hochberg) adjusted P-value smaller than 0.05. The raw sequence reads were normalized by size factors, then Log_2_(N + 1) transformed. Hierarchical clustering analysis was performed using the transformed reads, and a heat-map was drawn for the differentially expressed genes between the cisplatin treated cells and control cells, using the heatmap.2 [19] package of R. Function enrichment analysis was conducted based on PseudoCAP Function Class (http://www.pseudomonas.com), and a dot plots figure was generated using the ggplot2 [20] package of R.

The RNA-Seq datasets are available at the NCBI Sequence Read Archives: SRS1038085 and SRS1038089.

### qRT-PCR analysis

Total RNA was extracted using RNeasy Mini Kit (Qiagen) with on-column DNase digestion. The integrity, purity and concentration of the RNA were determined by NanoDrop spectrophotometry and Agilent 2200 TapeStation system.

Quantitative reverse transcriptase PCR (qRT-PCR) was performed using a two-step method. First-strand cDNA was synthesized from total RNA using SuperScript III First-Strand Synthesis SuperMix kit (Cat. No. 18080-400, Invitrogen). The cDNA was used as a template for qRT-PCR with a SYBR Select Master Mix kit (Cat. No. 4472953, Applied Biosystems by Life Technologies) on an Applied Biosystems StepOnePlus Real-Time PCR System with the specific primers (see Supporting Information File 1, Table S4). The three genes of GAPDH, *gyrB*, *rpoD* were used as endogenous control. Melting curve analyses were employed to verify the specific single-product amplification.

### *P. aeruginosa* killing assay

The OD_600_ of overnight cultures of PAO1, Δ*recA* and Δ*recA* complementation strains were measured and adjusted to 0.3 in ABTGC medium with 0, 3.125, 6.25 and 12.5 µM cisplatin, respectively. The strains were incubated at 37 °C with shaking at 200 rpm for 4 h. The cultures were then harvested, serially diluted and plated on LB agar plates for incubation at 37 °C overnight. Colonies on the plate were enumerated and colony forming units (CFU) mL^−1^ were tabulated as follows: CFU mL^−1^ = average number of colonies X dilution factor X volume used to spread on LB agar plate. Experiments were conducted in triplicate, and the results are shown as the mean ± s.d.

### *P. aeruginosa* biofilm killing assay by cisplatin and tobramycin

Biofilms were grown in 24-well plates (Nunc, Denmark) at 37 °C, as previously described [21]. Biofilms were washed 3 times with 0.9% NaCl and treated with ABTGC medium with 0, 3.125, 6.25 and 12.5 μM cisplatin, respectively. For tobramycin treatment, biofilms were treated with ABTGC medium with 5.3, 10.6 and 21.2 μM tobramycin, respectively. The treated biofilms were incubated at 37 °C for 4 h. The biofilms were harvested by scraping with a cell scraper, homogenized in 1 mL 0.9% NaCl, serially diluted and plated on LB agar plates for incubation at 37 °C overnight. Colonies on the plate were counted and CFU mL^−1^ was tabulated. Experiments were conducted in triplicate, and results are shown as the mean ± s.d.

### RAW264.7 macrophage cytotoxicity assay

The murine macrophages (RAW264.7) were maintained in Dulbecco’s modified Eagle’s medium (DMEM) (Life Technologies), supplemented with 10% fetal bovine serum (FBS) (Gibco), in 75 cm^2^ cell culture flasks at a density of 1.0 × 10^6^ cells mL^−1^ at 37 °C, 5% CO_2_ for 72 h. The 5.0 × 10^6^ macrophages per well were seeded in 24-well plate (Nunc, Denmark) and grown at 37 °C, 5% CO_2_ overnight. As previously described [12], macrophages were washed once with PBS and treated with PAO1 in DMEM medium with 0, 3.125, 6.25 and 12.5 µM cisplatin at a multiplicity of infection of 100 bacteria cells: 1 macrophage. As control, the macrophages were treated with the T3SS deficient Δ*pscJ* mutant in DMEM medium. The co-culture was incubated at 37 °C, 5% CO_2_ for 2 h. The extracellular bacterial cells in DMEM were removed and the infected macrophages were washed 3 times with PBS. Fresh DMEM was added to the infected macrophages and a further incubation of the macrophages at 37 °C, 5% CO_2_ for 4 h was ensued. A solution containing 20 µM propidium iodide (PI) was added to the macrophages to stain for dead macrophages killed by PAO1 treated with cisplatin or Δ*pscJ*. Live and dead macrophages were imaged by fluorescence microscopy (Zeiss, Germany) at 200× and tabulated under % of dead macrophages. Experiments were conducted in triplicate, and the results are shown as the mean ± s.d.

### Rabbit corneal wound healing model

New Zealand White Rabbits (*n* = 4, weighing 2 to 3 kg) purchased from National University of Singapore, were used for this study. All animal experiments were conducted in compliance with the ARVO statement for the Use of Animals in Ophthalmic and Vision Research, the guide for the Care and Use of laboratory animals (National Research Council) and under the supervision of Singhealth Experimental Medical Centre (SEMC).

Four rabbits were randomly grouped into two groups of two rabbits each, comprising of the PBS control treated group and 0.75 mg mL^–1^ cisplatin treated group. Intra peritoneal injection of 1 mL of ketamine (100 mg mL^−1^) and 0.5 mL of xylazil (20 mg mL^–1^) had been used to anesthetize the rabbits. Corneas were then anesthetized by topical administration of xylocaine 1%. A corneal wound was made by using a 5 mm trephine and mechanical removal of epithelial cells was carried out by sterile mini blade (BD-Beaver) leaving the basal lamina intact [22–23]. All the groups were treated by topical administration of the respective drug 3 times per day. Cornea wound was visualized by the aid of cobalt-blue filter equipped slit lamp biomicroscopy (New-generation Zoom clinical Slit Lamp, NS-2D, Righton), staining with Minims fluorescein sodium eye drops (Bausch and Lomb, 2% w/v) which is used in ophthalmology clinic for disclosure of wound on the ocular surface [24–25]. Residual wound area (pixel square) was measured during the wound healing process by Image-J 1.44o version. Mann-Whitney U Test was employed to determine if a difference in re-epithelialization existed among the different groups by using GraphPad Prism 6.02. A probability value of *p* ≤ 0.05 was considered statistically significant.

### Murine model for corneal infection

The corneas of the C57BL/6 mice were scratched with a scraper to create wounds. 10 µL of PAO1 planktonic cells (≈1 × 10^6^ cells) were dripped onto each cornea and incubated for 24 h on the mice to allow the biofilms to form on the scratched corneas as previously described [26–27]. About 10 µL of cisplatin (final concentration 25 µM) or 0.9% NaCl as control were dripped onto the cornea 3 times per day at 4 h interval on the second day. The mice were kept for 72 h and then sacrificed.

The corneas were harvested, and the biofilm was disrupted from the corneas by crushing with mortar and pestle, followed by vortexing with glass beads for 15 min. The homogenized biofilm cells were serially diluted, plated on LB agar plates and incubated at 37 °C overnight. The number of colonies was counted, and CFU ml^−1^ was tabulated. Experiments were performed in triplicate, and the results are shown as the mean ± s.d.

## Results and Discussion

### Cisplatin inhibits *P. aeruginosa* planktonic growth

**Figure 1:**
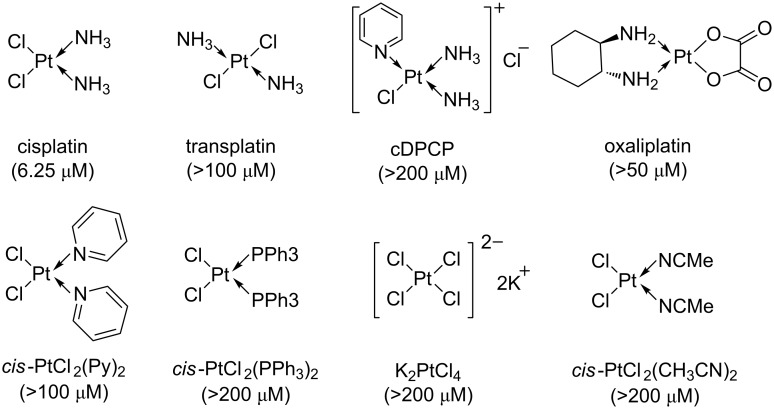
Structures and MICs of Pt-based compounds against *P. aeruginosa* PAO1.

During our screening of in-house collection of FDA-approved drugs against *P. aeruginosa* growth, we identified that cisplatin had a minimal inhibitory concentration (MIC) of 6.25 μM against the *P. aeruginosa* PAO1 lab strain (Figure 1). We further tested the growth inhibitory effect of cisplatin against another *P. aeruginosa* lab strain PA14 and a *P. aeruginosa* mucoid multiple-drug resistant (MDR) CF clinical isolate 57388A and found that cisplatin had equivalent MIC at 6.25 μM against these two strains (Supporting Information File 1, Table S3). We next evaluated several other Pt(II)-based compounds for their growth inhibitory effects on *P. aeruginosa*, but these tested compounds had higher MIC against *P. aeruginosa* as compared to cisplatin (Figure 1).

### Mode of action

**Figure 2:**
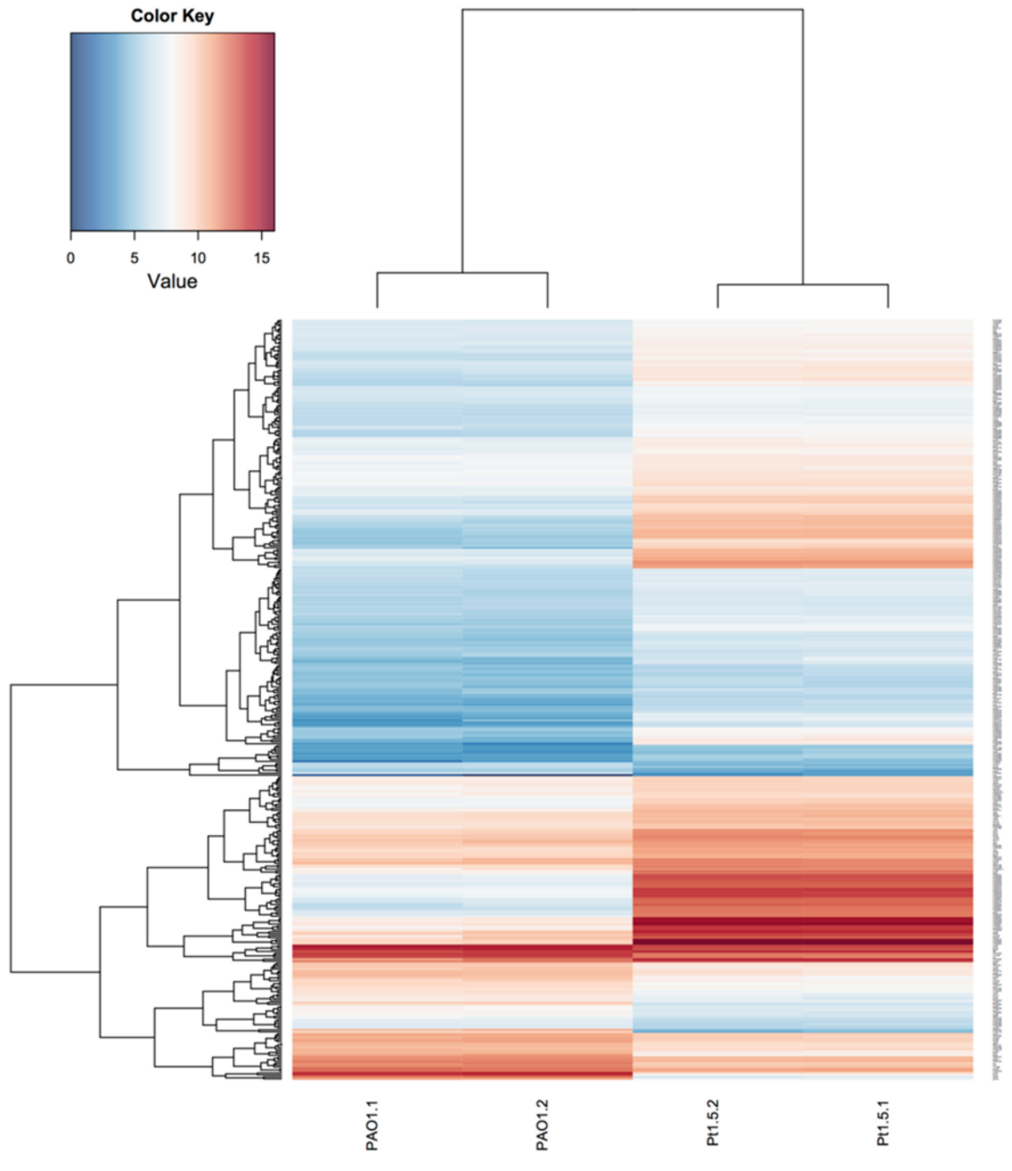
Transcriptomic analysis of control and cisplatin-treated PAO1 cultures. Heatmap comparing the transcriptomes of control and cisplatin-treated PAO1 cultures.

**Figure 3:**
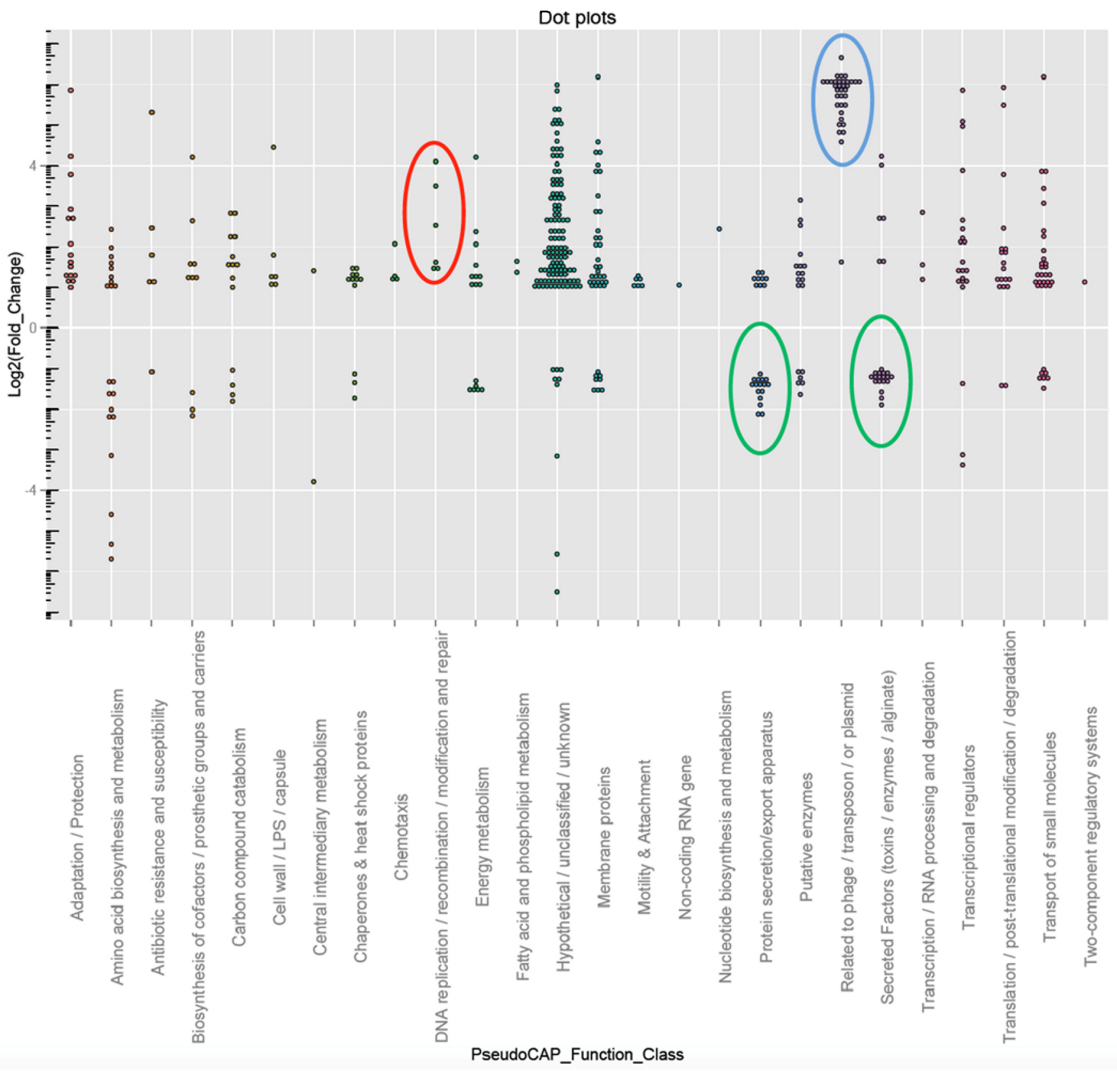
Function enrichment of differentially expressed genes from the transcriptomic analysis. A dot-lot figure was generated using ggplot2 the package of R. Red circle highlights the genes involved in DNA replication and repair; blue circle highlights the genes involved in pyocin synthesis; green circle highlights the genes involved in protein secretion (T3SS).

The growth inhibitory effects of cisplatin on both eukaryotic cells and microbial cells are attributed to the interactions of Pt(II) in cisplatin with DNA [28–30]. To reveal the growth arresting mechanisms and overall impact of cisplatin on the physiology of *P. aeruginosa*, we performed an RNA-sequencing (RNA-seq) based transcriptomic analysis on *P. aeruginosa* PAO1 after cultivation in sub-lethal concentration (1.5 µM) of cisplatin for 8 hours and compared the transcriptome with control transcriptomes of bacteria present in cisplatin-free medium. Using a negative binomial test with a BH adjusted P-value cut-off of 0.05 and a fold-change cut-off of 2, we found that sub-MIC cisplatin treatment induced the expression of 315 genes (Supporting Information File 1, Table S1) while repressed the expression of 72 genes (Supporting Information File 1, Table S2) in *P. aeruginosa*. The heat-map and function enrichment of the genes that were differently expressed between cisplatin-treated and control *P. aeruginosa* samples were illustrated in Figure 2 and Figure 3, respectively.

The cisplatin treatment triggered the expression of a large fraction of the LexA-controlled SOS regulon [31], including genes involved in DNA replication, recombination, modification and repair (*dnaE2*, *imuB*, *imuA*, *dinG*, *recA*, *recN*, *recX*) and genes involved in pyocin synthesis (*PA0614*-*PA0648*), whose expression were previously reported to be induced by ciprofloxacin [31] and hydrogen peroxide treatments [32]. In addition, cisplatin treatment induced the expression of a series of genes involved in energy metabolism, which corroborated with previous proteomics work showing that cisplatin could interfere with stress response and energy metabolism in *E. coli* [33].

**Figure 4:**
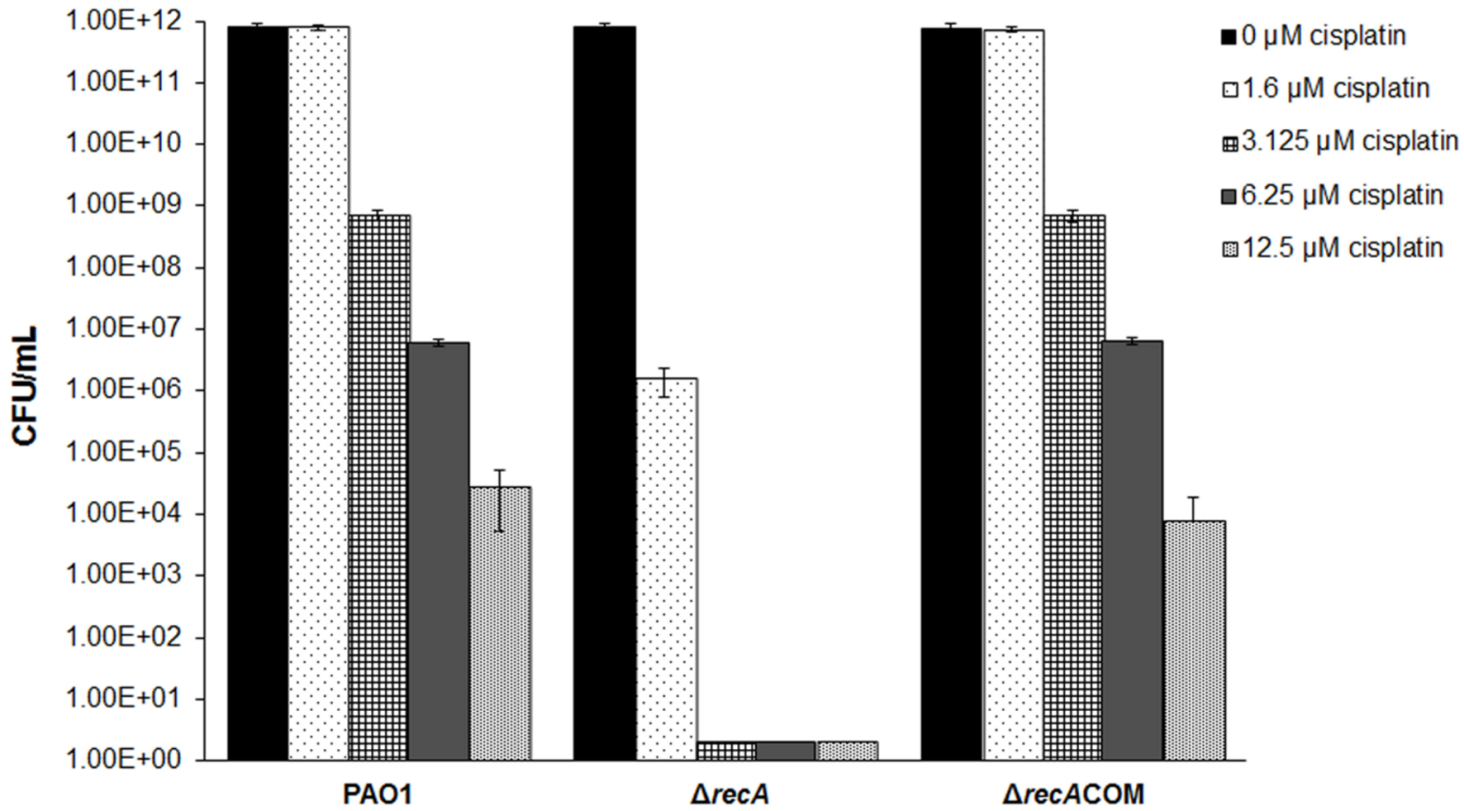
Cisplatin fast-kill assay against the *P. aeruginosa* PAO1, Δ*recA* mutant and the Δ*recA*COM strain. *P. aeruginosa* strains were treated by ABTGC medium with varies concentrations of cisplatin for 4 h. CFUs were determined for cisplatin-treated *P. aeruginosa* cultures. Means and s.d. from triplicate experiments are shown.

To further validate the impact of cisplatin on DNA replication, we compared the cisplatin sensitivity of the *P. aeruginosa* wild-type PAO1 strain and its DNA recombination-deficient *recA* mutant and found that the *rec* recombination pathway was essential for the cisplatin resistance in *P. aeruginosa* (Figure 4). Together with the transcriptome profiling, this result confirmed that cisplatin was able to interact with the *P. aeruginosa* DNA, resulting in up-regulation of stress response genes. This mechanism was also similar to the mechanism of action by another DNA crosslinker, mitomycin C which kills bacterial persister cells [34].

### Anti-T3SS effect of cisplatin

**Figure 5:**
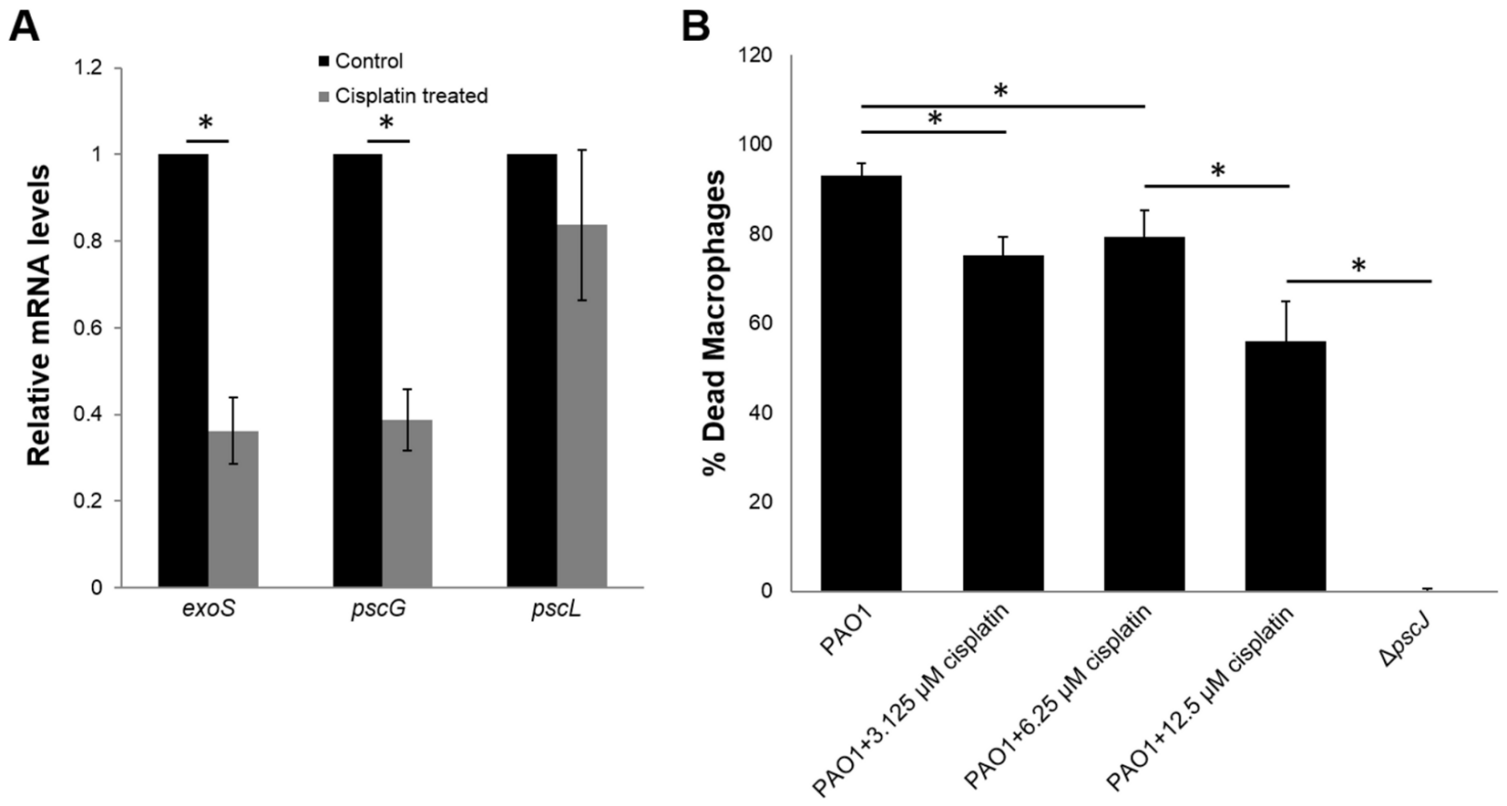
Cisplatin treatment represses T3SS associated virulence. (A) Cisplatin treatment downregulated the expression of T3SS gene revealed by qRT-PCR analysis. Means and s.d. from triplicate experiments are shown. Student’s t-test was performed for testing differences between groups. *P ≤ 0.05. (B) Cisplatin treatment reduced cytotoxicity of *P. aeruginosa* PAO1 against mouse macrophage cells. Means and s.d. from triplicate experiments are shown. Student’s t-test was performed for testing differences between groups. *P ≤ 0.05.

Our transcriptomic analysis also revealed the expression of a large number of the secretion related genes, including those of the type III secretion system (T3SS), which were downregulated in PAO1 by cisplatin exposure (Table S2), which was similar to that by ciprofloxacin exposure [31]. Our qRT-PCR analysis confirmed that the expression of two selected T3SS genes, *exoS* and *pscG*, were downregulated by cisplatin treatment compared to control (Figure 5a). The downregulation of T3SS by the LexA-controlled SOS response [35] could be attributed to the induced expression of *ptrB*, a repressor of T3SS by cisplatin treatment (Supporting Information File 1, Table S3).

The T3SS is one of the major virulence mechanisms employed by *P. aeruginosa* and other microbial pathogens to impair the host immune systems during infection [36–37]. T3SS activity of *P. aeruginosa* was correlated with acute cytotoxicity to host epithelial cells and immune cells such as macrophages and neutrophils [38]. As we demonstrated that cisplatin treatment was able to reduce the T3SS of *P. aeruginosa*, we further tested the ability of cisplatin in attenuating the acute cytotoxicity of *P. aeruginosa* to macrophages. Cisplatin treatment of *P. aeruginosa* in the *P. aeruginosa*-macrophage co-cultures caused significant less death of the mouse macrophages compared to control samples (Figure 5b), suggesting the effectiveness of cisplatin against *P. aeruginosa* infection.

### Antibiofilm effect of cisplatin

**Figure 6:**
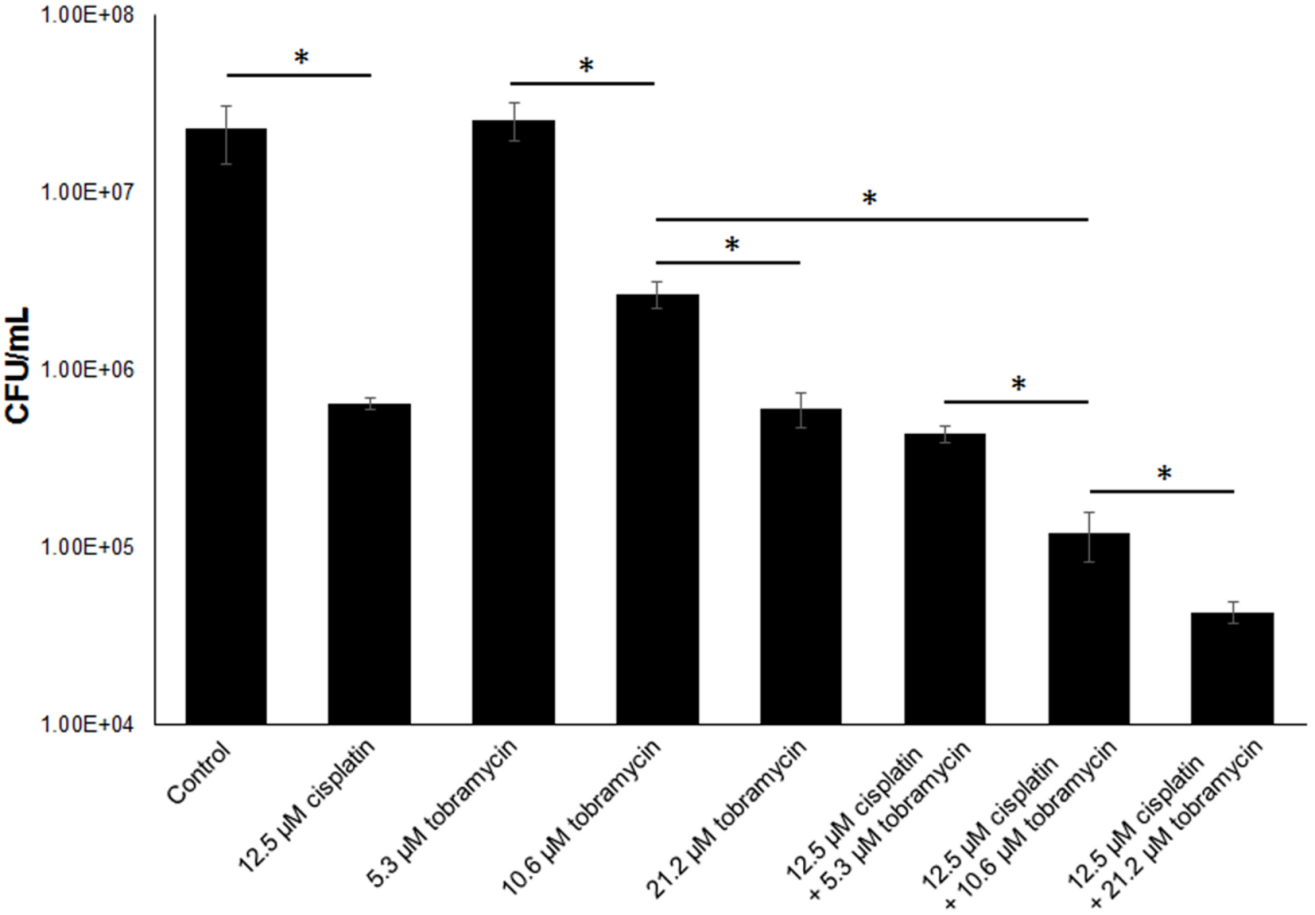
*P. aeruginosa* biofilm killing assay by cisplatin, tobramycin and their combinations. *P. aeruginosa* biofilms were treated by ABTGC medium with varies concentrations of cisplatin and or tobramycin for 4 h. CFUs were determined for cisplatin and or tobramycin-treated *P. aeruginosa* biofilms. Means and s.d. from triplicate experiments are shown. Student’s t-test was performed for testing differences between groups. *P ≤ 0.05.

*P. aeruginosa* is notorious for its biofilm formation capacity, which might lead to persistent or recalcitrant infections. SOS response and DNA recombination are required for development of *P. aeruginosa* biofilm resistance [39–41]. Given cisplatin treatment was able to interfere with DNA repair, we hypothesized that cisplatin treatment could eradicate *P. aeruginosa* biofilm cells. We compared the biofilm killing effects of cisplatin and tobramycin at various concentrations. The MIC of cisplatin and tobramycin against planktonic *P. aeruginosa* cells were 6.25 µM and 2.65 µM, respectively. However, tobramycin could not kill the biofilm cells at 2 × MIC due to its limitation in biofilm penetration [42], while cisplatin was able to kill substantial amount of biofilm cells with nearly 100 times reduction of *P. aeruginosa* biofilm cells (Figure 6). This result suggested that cisplatin might penetrate the biofilms better than the otherwise eDNA trapped tobramycin to kill the *P. aeruginosa* cells [42]. The 4 × MIC and 8 × MIC of tobramycin treatment showed dose-dependent increase of biofilm killing capacity (Figure 6). Interestingly, cisplatin had combinatory effects with tobramycin in killing the PAO1 biofilms, as combinatorial treatment of 2 × MIC of cisplatin with 4 × MIC or 8 × MIC tobramycin killed the biofilm cells at a higher rate compared to the mono-compound treatment (Figure 6). These results suggest that combination of cisplatin and other conventional antimicrobials could be a useful strategy for eradicating persistent biofilm-associated infections.

### Cisplatin treatment attenuates *P. aeruginosa* infection

**Figure 7:**
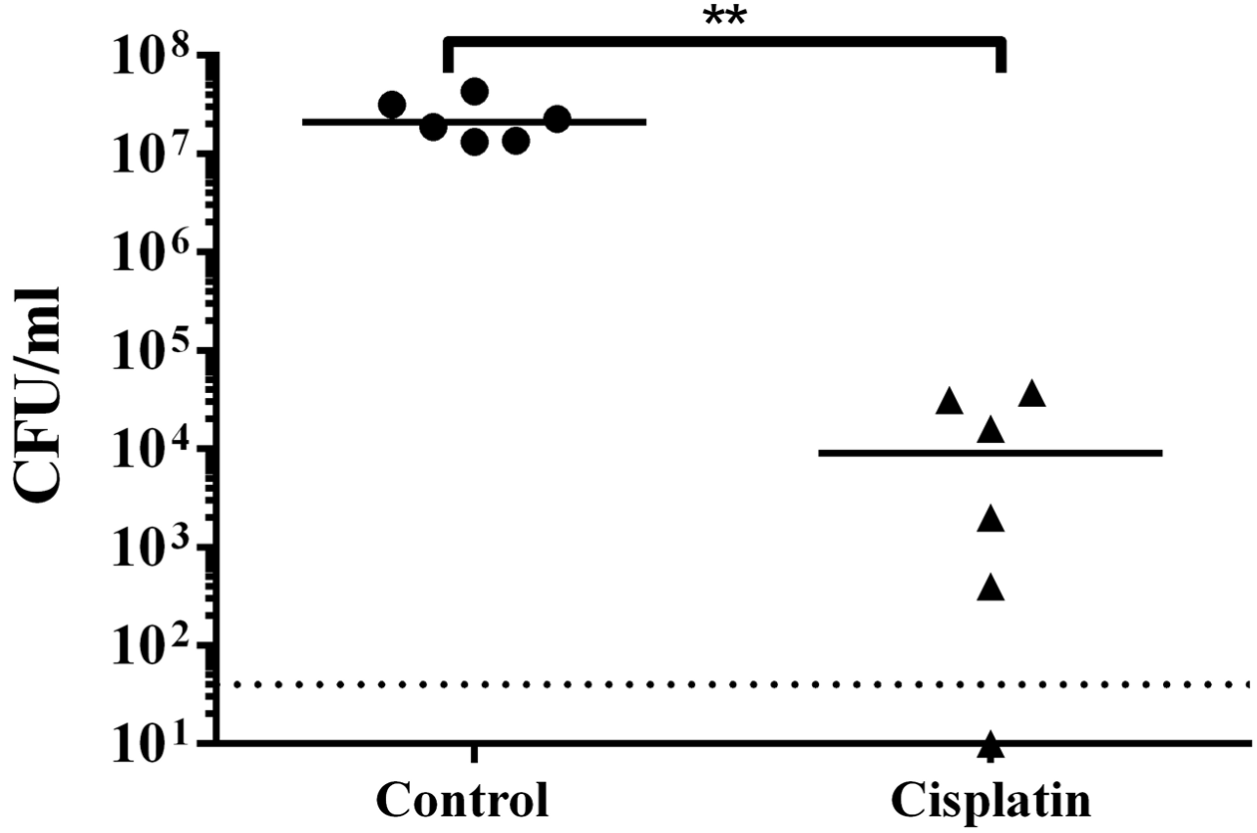
Cisplatin treatment attenuates *P. aeruginosa* infections. CFU mL^−1^ of PAO1 obtained from corneas with and without cisplatin treatment. Dotted horizontal lines represent limit of detection. The mean and s.d. from six experiments were shown for in vivo biofilms. Student’s t-test was performed for testing differences between groups. *P < 0.01.

As cisplatin could reduce the synthesis of T3SS-mediated virulent products and kill biofilms of *P. aeruginosa*, we further tested if cisplatin treatment was able to eradicate in vivo *P. aeruginosa* infections using a mouse model of keratitis, where *P. aeruginosa* cells have biofilm-like morphology [26–27] and employ type III secretion during infections [43]. We firstly confirmed that cisplatin was not toxic and did not interfere with wound healing, with no observable inflammation or adverse effects, when applied topically on scratched corneas with no bacterial infection (Supporting Information File 1, Figure S1). We then allowed *P. aeruginosa* PAO1 to colonize and establish infection in the scratched corneas of mice for 24 h. 10 µL of 1 × MIC (6.25 µM) of cisplatin and control 0.9% NaCl were dripped at the site of *P. aeruginosa* infection 3 times (4 hour interval) on the second day. The mice were sacrificed on the third day and their corneas were harvested for CFU count. Cisplatin showed efficient killing capacity on *P. aeruginosa* cells from infected mouse corneas and there was a significant reduction in the bacterial loads from the cisplatin treated corneas as compared to the control corneas (Figure 7).

## Conclusion

Here, we have demonstrated how cisplatin displays antivirulence and antibiofilm effects against the opportunistic pathogen *P. aeruginosa*. Since biofilms are notoriously difficult to be cleared by conventional antibiotics, cisplatin possesses the additional advantage of killing biofilms. This makes cisplatin a more attractive antimicrobial for treating biofilm infections clinically. Even though cisplatin is known for its toxic side effects on cancer patients when administered intravenously, we showed indications that cisplatin could be applied topically to infection sites with low toxicity and minimal negative impact on wound repair. Transcriptomic analysis revealed that the working mechanism of cisplatin towards *P. aeruginosa* is rather unique and distinct from other conventional antibiotics, which may offer alternative therapeutic approaches towards persistent infections.

In recent years, metal-containing compounds have been identified as antimicrobial agents. Gallium was shown to disrupt the iron metabolism of *P. aeruginosa* and efficiently kill established biofilm [44]. In addition, the gold-containing drug, auranofin, was found to be a broad-spectrum bactericidal compound, that targets the thiol-redox homeostasis of a range of Gram-positive bacteria [45]. Further studies will be carried out to better understand the resistance mechanism and structural requirements of the Pt-based compounds as an alternative to the conventional antibiotics. Such compounds could also be used synergistically with specific enzymes that degrade the biofilm matrix [46] or biofilm-dispersal agents to boost the eradication of biofilms, to provide better treatment options for chronic and persistent infections.

## Supporting Information

File 1 Additional information.
