# Supplementary material for: Repurposing the anticancer drug cisplatin with the aim of developing novel Pseudomonas aeruginosa infection control agents
Source: Beilstein J Org Chem. 2018 Dec 14;14:3059–69. doi: 10.3762/bjoc.14.284 (PMC6296412; doi:10.3762/bjoc.14.284)
Supplement: File 1 — Additional information. [file Beilstein_J_Org_Chem-14-3059-s001.pdf]

BEILSTEIN JOURNAL OF ORGANIC CHEMISTRY

Supporting Information

for

Repurposing the anticancer drug cisplatin with the aim of developing novel *Pseudomonas aeruginosa* infection control agents

Mingjun Yuan, Song Lin Chua, Yang Liu, Daniela I. Drautz-Moses,
Joey Kuok Hoong Yam, Thet Tun Aung, Roger W. Beuerman,
May Margarette Santillan Salido, Stephan C. Schuster, Choon-Hong Tan,
Michael Givskov, Liang Yang and Thomas E. Nielsen

Beilstein J. Org. Chem. **2018,** *14,* 3059–3069. doi:10.3762/bjoc.14.284

Additional information

License and Terms: This is a supporting information file under the terms of the Creative Commons Attribution License (http://creativecommons.org/licenses/by/4.0). Please note that the reuse, redistribution and reproduction in particular requires that the authors and source are credited.
The license is subject to the *Beilstein Journal of Organic Chemistry* terms and conditions: (https://www.beilstein-journals.org/bjoc)

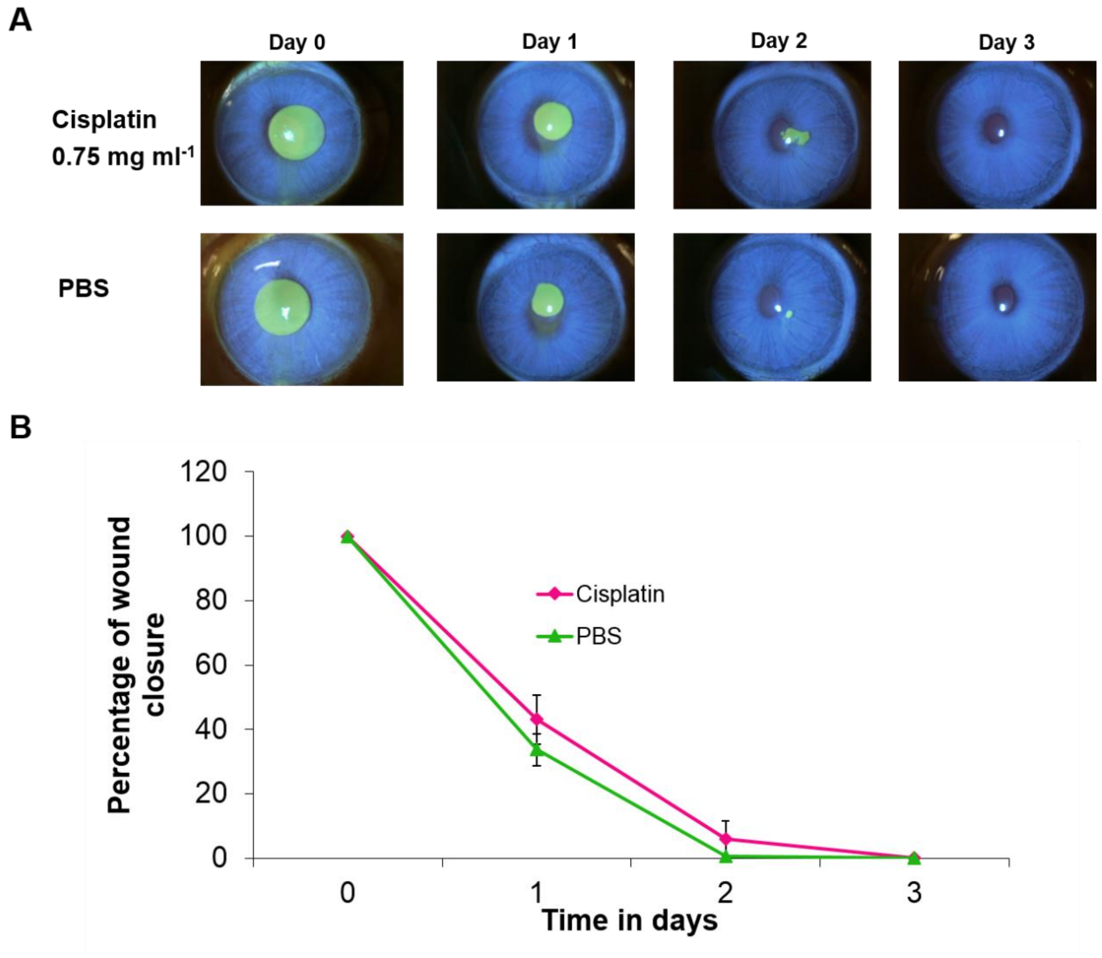

Figure S1: Rabbit corneal wound healing model. (a) Recovery and closure of wound with cisplatin treatment. (b) Percentage of wound closure on the scratched cornea.

Table S1: Genes induced at least 2 folds in PAO1 by cisplatin treatment.

Locus_Tag	fold change	P-Value	Gene name	Annnotation
PA0045	2.0	0.0011	PA0045	Hypothetical, unclassified, unknown
PA0046	3.1	0.0001	PA0046	Hypothetical, unclassified, unknown
PA0069	21.8	0.0000	PA0069	Hypothetical, unclassified, unknown
PA0070	2.1	0.0000	PA0070	Membrane proteins; Protein secretion/export apparatus
PA0083	2.3	0.0000	PA0083	Protein secretion/export apparatus
PA0084	2.4	0.0000	PA0084	Protein secretion/export apparatus
PA0085	3.2	0.0000	hcp1	Secreted Factors (toxins, enzymes, alginate)
PA0086	2.5	0.0000	PA0086	Protein secretion/export apparatus
PA0087	2.6	0.0020	PA0087	Protein secretion/export apparatus
PA0088	2.1	0.0000	PA0088	Protein secretion/export apparatus
PA0089	2.3	0.0000	PA0089	Protein secretion/export apparatus
PA0090	2.4	0.0000	clpV1	Translation, post-translational modification, degradation; Chaperones & heat shock proteins; Protein secretion/export apparatus
PA0091	2.0	0.0000	vgrG1	Protein secretion/export apparatus
PA0126	2.9	0.0000	PA0126	Hypothetical, unclassified, unknown
PA0151	2.4	0.0000	PA0151	Transport of small molecules
PA0277	3.6	0.0000	PA0277	Hypothetical, unclassified, unknown
PA0320	3.6	0.0000	PA0320	Hypothetical, unclassified, unknown
PA0514	18.4	0.0441	nirL	Hypothetical, unclassified, unknown; Energy metabolism; Biosynthesis of cofactors, prosthetic groups and carriers
PA0516	2.4	0.0129	nirF	Energy metabolism; Biosynthesis of cofactors, prosthetic groups and carriers
PA0528	2.7	0.0001	PA0528	Transcriptional regulators
PA0578	2.2	0.0000	PA0578	Hypothetical, unclassified, unknown
PA0608	2.9	0.0000	PA0608	Carbon compound catabolism
PA0610	34.0	0.0000	prtN	Transcriptional regulators
PA0611	4.5	0.0000	prtR	Transcriptional regulators
PA0612	31.3	0.0000	ptrB	Transcriptional regulators
PA0613	41.5	0.0000	PA0613	Hypothetical, unclassified, unknown
PA0614	63.6	0.0000	PA0614	Hypothetical, unclassified, unknown
PA0615	42.3	0.0000	PA0615	Hypothetical, unclassified, unknown
PA0616	66.3	0.0000	PA0616	Related to phage, transposon, or plasmid
PA0617	61.7	0.0000	PA0617	Related to phage, transposon, or plasmid
PA0618	67.4	0.0000	PA0618	Related to phage, transposon, or plasmid
PA0619	72.7	0.0000	PA0619	Related to phage, transposon, or plasmid
PA0620	51.7	0.0000	PA0620	Related to phage, transposon, or plasmid
PA0621	43.7	0.0000	PA0621	Related to phage, transposon, or plasmid
PA0622	64.5	0.0000	PA0622	Related to phage, transposon, or plasmid
PA0623	67.0	0.0000	PA0623	Related to phage, transposon, or plasmid
PA0624	66.1	0.0000	PA0624	Related to phage, transposon, or plasmid
PA0625	57.4	0.0000	PA0625	Related to phage, transposon, or plasmid

PA0626	52.9	0.0000	PA0626	Related to phage, transposon, or plasmid
PA0627	46.3	0.0000	PA0627	Related to phage, transposon, or plasmid
PA0628	62.7	0.0000	PA0628	Related to phage, transposon, or plasmid
PA0629	60.5	0.0000	PA0629	Related to phage, transposon, or plasmid
PA0630	32.7	0.0000	PA0630	Related to phage, transposon, or plasmid
PA0631	46.4	0.0000	PA0631	Related to phage, transposon, or plasmid
PA0632	56.6	0.0000	PA0632	Related to phage, transposon, or plasmid
PA0633	67.8	0.0000	PA0633	Related to phage, transposon, or plasmid
PA0634	66.2	0.0000	PA0634	Related to phage, transposon, or plasmid
PA0635	61.1	0.0000	PA0635	Related to phage, transposon, or plasmid
PA0636	68.8	0.0000	PA0636	Related to phage, transposon, or plasmid
PA0637	75.6	0.0000	PA0637	Related to phage, transposon, or plasmid
PA0638	71.3	0.0000	PA0638	Related to phage, transposon, or plasmid
PA0640	68.4	0.0000	PA0640	Related to phage, transposon, or plasmid
PA0641	65.8	0.0000	PA0641	Related to phage, transposon, or plasmid
PA0642	100.6	0.0000	PA0642	Related to phage, transposon, or plasmid
PA0643	53.8	0.0000	PA0643	Related to phage, transposon, or plasmid
PA0644	31.7	0.0000	PA0644	Related to phage, transposon, or plasmid
PA0645	39.4	0.0000	PA0645	Related to phage, transposon, or plasmid
PA0646	35.2	0.0000	PA0646	Related to phage, transposon, or plasmid
PA0647	28.1	0.0000	PA0647	Related to phage, transposon, or plasmid
PA0648	28.3	0.0000	PA0648	Related to phage, transposon, or plasmid
PA0649	2.4	0.0000	trpG	anthranilate synthase component II
PA0654	2.7	0.0000	speD	S-adenosylmethionine decarboxylase proenzyme
PA0669	5.7	0.0000	dnaE2	Putative enzymes; DNA replication, recombination, modification and repair
PA0670	9.2	0.0000	imuB	DNA polymerase-like protein PA0670
PA0671	18.4	0.0000	imuA	Predicted RecA/RadA recombinase
PA0729	2.2	0.0000	PA0729	Hypothetical, unclassified, unknown
PA0730	2.9	0.0000	PA0730	Putative enzymes
PA0731	2.6	0.0000	PA0731	Hypothetical, unclassified, unknown
PA0733	2.3	0.0000	PA0733	Transcription, RNA processing and degradation
PA0780	2.3	0.0000	pruR	proline utilization regulator
PA0781	33.8	0.0000	PA0781	Hypothetical, unclassified, unknown
PA0805	2.6	0.0000	PA0805	Hypothetical, unclassified, unknown
PA0807	39.8	0.0000	ampDh3	Antibiotic resistance and susceptibility
PA0808	9.6	0.0000	PA0808	Hypothetical, unclassified, unknown
PA0809	14.7	0.0000	PA0809	Membrane proteins; Transport of small molecules
PA0810	6.9	0.0000	PA0810	Carbon compound catabolism
PA0811	14.5	0.0000	PA0811	Membrane proteins; Transport of small molecules
PA0812	20.7	0.0000	PA0812	Hypothetical, unclassified, unknown
PA0813	9.5	0.0000	PA0813	Hypothetical, unclassified, unknown
PA0814	9.8	0.0000	PA0814	Hypothetical, unclassified, unknown

PA0815	2.7	0.0000	PA0815	Transcriptional regulators
PA0816	6.3	0.0000	PA0816	Transcriptional regulators
PA0817	8.9	0.0000	PA0817	Putative enzymes
PA0818	3.8	0.0000	PA0818	Hypothetical, unclassified, unknown
PA0819	10.2	0.0000	PA0819	Hypothetical, unclassified, unknown
PA0821	2.3	0.0000	PA0821	Hypothetical, unclassified, unknown
PA0822	2.8	0.0000	PA0822	Hypothetical, unclassified, unknown
PA0823	3.5	0.0000	PA0823	Hypothetical, unclassified, unknown
PA0824	6.4	0.0000	PA0824	Hypothetical, unclassified, unknown
PA0825	11.8	0.0000	PA0825	Hypothetical, unclassified, unknown
PA0826	4.7	0.0000	PA0826	Hypothetical, unclassified, unknown
PA0827	3.4	0.0000	PA0827	Hypothetical, unclassified, unknown
PA0828	4.2	0.0000	PA0828	Transcriptional regulators
PA0829	6.3	0.0000	PA0829	Putative enzymes
PA0830	3.6	0.0000	PA0830	Hypothetical, unclassified, unknown
PA0831	2.3	0.0000	oruR	transcriptional regulator OruR
PA0834	4.6	0.0000	PA0834	Hypothetical, unclassified, unknown
PA0839	2.6	0.0007	PA0839	Transcriptional regulators
PA0840	2.4	0.0000	PA0840	Putative enzymes
PA0842	3.5	0.0000	PA0842	Putative enzymes
PA0844	3.1	0.0000	plcH	Secreted Factors (toxins, enzymes, alginate)
PA0845	3.3	0.0000	PA0845	Hypothetical, unclassified, unknown
PA0848	2.0	0.0024	PA0848	Adaptation, Protection; Putative enzymes
PA0865	3.9	0.0000	hpd	4-hydroxyphenylpyruvate dioxygenase
PA0872	2.2	0.0000	phhA	phenylalanine-4-hydroxylase
PA0906	4.6	0.0000	PA0906	Transcriptional regulators
PA0907	15.0	0.0000	PA0907	Hypothetical, unclassified, unknown
PA0908	31.7	0.0000	PA0908	Hypothetical, unclassified, unknown
PA0909	23.9	0.0000	PA0909	Related to phage, transposon, or plasmid; Membrane proteins
PA0910	34.5	0.0000	PA0910	Hypothetical, unclassified, unknown
PA0911	32.6	0.0000	PA0911	Hypothetical, unclassified, unknown
PA0912	19.4	0.0000	PA0912	Hypothetical, unclassified, unknown
PA0913	3.0	0.0000	mgtE	Transport of small molecules
PA0914	6.3	0.0000	PA0914	Hypothetical, unclassified, unknown
PA0915	8.4	0.0000	PA0915	Hypothetical, unclassified, unknown
PA0916	2.9	0.0000	PA0916	Hypothetical, unclassified, unknown
PA0918	5.2	0.0000	PA0918	cytochrome b561
PA0922	12.4	0.0000	PA0922	Hypothetical, unclassified, unknown
PA0923	3.1	0.0000	dinB	Adaptation, Protection; DNA replication, recombination, modification and repair
PA0924	2.6	0.0000	PA0924	Hypothetical, unclassified, unknown; Membrane proteins
PA0939	2.0	0.0106	PA0939	Hypothetical, unclassified, unknown
PA0977	3.9	0.0000	PA0977	Hypothetical, unclassified, unknown

PA0978	3.1	0.0000	PA0978	Related to phage, transposon, or plasmid
PA0985	16.2	0.0000	pyoS5	Membrane proteins; Secreted Factors (toxins, enzymes, alginate)
PA1044	2.4	0.0001	PA1044	Membrane proteins
PA1045	2.6	0.0000	dinG	ATP-dependent helicase DinG/Rad3
PA1077	2.4	0.0000	flgB	flagellar basal-body rod protein FlgB
PA1078	2.1	0.0000	flgC	flagellar basal-body rod protein FlgC
PA1081	2.1	0.0000	flgF	flagellar basal-body rod protein FlgF
PA1092	2.0	0.0000	fliC	flagellin type B
PA1096	2.1	0.0000	PA1096	Hypothetical, unclassified, unknown
PA1100	2.4	0.0000	fliE	flagellar hook-basal body complex protein FliE
PA1150	18.6	0.0000	pys2	pyocin S2
PA1151	7.6	0.0000	imm2	pyocin S2 immunity protein
PA1152	9.4	0.0000	PA1152	Hypothetical, unclassified, unknown
PA1153	2.7	0.0321	PA1153	Hypothetical, unclassified, unknown
PA1178	2.2	0.0000	oprH	PhoP/Q and low Mg2+ inducible outer membrane protein H1 precursor
PA1326	2.1	0.0308	ilvA2	"threonine dehydratase, biosynthetic"
PA1331	2.4	0.0001	PA1331	Hypothetical, unclassified, unknown
PA1435	3.0	0.0000	PA1435	Transport of small molecules
PA1466	7.9	0.0000	PA1466	Hypothetical, unclassified, unknown
PA1546	3.0	0.0000	hemN	oxygen-independent coproporphyrinogen III oxidase
PA1547	4.5	0.0000	PA1547	Membrane proteins
PA1548	2.7	0.0000	PA1548	Hypothetical, unclassified, unknown
PA1549	4.8	0.0000	PA1549	Membrane proteins; Transport of small molecules
PA1550	2.0	0.0014	PA1550	Hypothetical, unclassified, unknown
PA1551	2.1	0.0000	PA1551	Energy metabolism
PA1591	2.1	0.0277	PA1591	Membrane proteins
PA1596	2.3	0.0000	htpG	heat shock protein HtpG
PA1608	2.4	0.0000	PA1608	Adaptation, Protection; Chemotaxis
PA1679	2.3	0.0000	PA1679	Hypothetical, unclassified, unknown
PA1791	2.0	0.0004	PA1791	Hypothetical, unclassified, unknown
PA1797	2.3	0.0000	PA1797	Hypothetical, unclassified, unknown
PA1798	2.2	0.0000	parS	Two-component regulatory systems; Antibiotic resistance and susceptibility
PA1845	3.9	0.0003	PA1845	Hypothetical, unclassified, unknown
PA1854	2.2	0.0001	PA1854	Hypothetical, unclassified, unknown
PA1865	4.7	0.0000	PA1865	Hypothetical protein, restriction endonuclease-like VRR-NUC domain
PA1866	2.0	0.0000	PA1866	Rad3-related DNA helicases
PA1892	3.1	0.0000	PA1892	Hypothetical, unclassified, unknown
PA1921	2.0	0.0034	PA1921	Hypothetical, unclassified, unknown
PA1922	10.9	0.0000	PA1922	Transport of small molecules
PA1923	6.2	0.0000	PA1923	Biosynthesis of cofactors, prosthetic groups and carriers
PA1924	3.4	0.0357	PA1924	Hypothetical, unclassified, unknown

PA1925	4.0	0.0000	PA1925	Hypothetical, unclassified, unknown
PA1959	3.5	0.0000	bacA	bacitracin resistance protein
PA1967	2.0	0.0032	PA1967	Hypothetical, unclassified, unknown
PA1999	3.4	0.0000	dhcA	Amino acid biosynthesis and metabolism; Carbon compound catabolism
PA2000	3.0	0.0000	dhcB	Amino acid biosynthesis and metabolism; Carbon compound catabolism
PA2009	2.3	0.0000	hmgA	"homogentisate 1,2-dioxygenase"
PA2012	3.0	0.0000	liuD	Carbon compound catabolism
PA2013	4.9	0.0000	liuC	Carbon compound catabolism
PA2014	4.6	0.0000	liuB	Carbon compound catabolism
PA2015	7.2	0.0000	liuA	Carbon compound catabolism
PA2016	5.4	0.0000	liuR	Transcriptional regulators
PA2036	2.7	0.0395	PA2036	Hypothetical, unclassified, unknown
PA2258	2.0	0.0046	ptxR	transcriptional regulator PtxR
PA2278	2.5	0.0007	arsB	ArsB protein
PA2282	5.3	0.0000	PA2282	Hypothetical, unclassified, unknown
PA2283	8.9	0.0000	PA2283	Hypothetical, unclassified, unknown
PA2284	6.4	0.0000	PA2284	Hypothetical, unclassified, unknown
PA2285	11.5	0.0000	PA2285	Hypothetical, unclassified, unknown
PA2286	19.5	0.0000	PA2286	Membrane proteins
PA2287	24.2	0.0000	PA2287	Hypothetical, unclassified, unknown
PA2288	35.0	0.0000	PA2288	Hypothetical, unclassified, unknown
PA2428	2.8	0.0000	PA2428	Hypothetical, unclassified, unknown
PA2439	2.2	0.0089	PA2439	Membrane proteins
PA2493	2.2	0.0003	mexE	Resistance-Nodulation-Cell Division (RND) multidrug efflux membrane fusion protein MexE precursor
PA2554	2.5	0.0000	PA2554	Putative enzymes
PA2555	2.9	0.0000	PA2555	Putative enzymes
PA2557	2.6	0.0000	PA2557	Fatty acid and phospholipid metabolism
PA2560	2.5	0.0000	PA2560	Hypothetical, unclassified, unknown
PA2654	2.3	0.0000	PA2654	Adaptation, Protection; Chemotaxis
PA2767	2.2	0.0003	PA2767	Putative enzymes
PA2768	2.9	0.0008	PA2768	Membrane proteins
PA2840	7.2	0.0000	PA2840	Transcription, RNA processing and degradation
PA2926	2.8	0.0209	hisP	histidine transport protein HisP
PA2970	2.0	0.0000	rpmF	50S ribosomal protein L32
PA2971	2.3	0.0000	PA2971	Hypothetical, unclassified, unknown
PA3007	13.8	0.0000	lexA	repressor protein LexA
PA3008	12.9	0.0000	PA3008	Hypothetical, unclassified, unknown
PA3009	2.4	0.0000	PA3009	Hypothetical, unclassified, unknown
PA3126	2.4	0.0000	ibpA	heat-shock protein IbpA
PA3174	3.1	0.0000	PA3174	Transcriptional regulators
PA3175	2.1	0.0126	PA3175	Amino acid biosynthesis and metabolism

PA3176	2.2	0.0175	gltS	sodium/glutamate symporter GltS
PA3268	2.2	0.0000	PA3268	Membrane proteins; Transport of small molecules
PA3391	2.2	0.0020	nosR	regulatory protein NosR
PA3392	2.4	0.0000	nosZ	nitrous-oxide reductase precursor
PA3393	2.0	0.0216	nosD	NosD protein
PA3395	4.2	0.0000	nosY	NosY protein
PA3396	2.9	0.0165	nosL	NosL protein
PA3413	4.5	0.0000	yebG	SOS regulon DNA damage-inducible protein
PA3414	3.0	0.0000	PA3414	Hypothetical, unclassified, unknown
PA3526	2.2	0.0000	PA3526	Membrane proteins
PA3532	2.4	0.0087	PA3532	Membrane proteins
PA3597	2.1	0.0002	PA3597	Membrane proteins; Transport of small molecules
PA3598	3.4	0.0000	PA3598	Hypothetical, unclassified, unknown
PA3599	14.8	0.0000	PA3599	Transcriptional regulators
PA3600	45.0	0.0000	PA3600	Translation, post-translational modification, degradation
PA3601	60.9	0.0000	PA3601	Translation, post-translational modification, degradation
PA3610	2.1	0.0216	potD	polyamine transport protein PotD
PA3616	6.2	0.0000	recX	Regulatory protein RecX
PA3617	11.3	0.0000	recA	RecA protein
PA3661	4.0	0.0000	PA3661	Hypothetical, unclassified, unknown
PA3677	2.1	0.0160	PA3677	Transport of small molecules
PA3727	6.3	0.0000	PA3727	Hypothetical, unclassified, unknown
PA3728	5.4	0.0000	PA3728	Hypothetical, unclassified, unknown
PA3729	6.8	0.0000	PA3729	Hypothetical, unclassified, unknown
PA3730	7.3	0.0000	PA3730	Membrane proteins
PA3731	6.2	0.0000	PA3731	Hypothetical, unclassified, unknown
PA3732	7.3	0.0000	PA3732	Hypothetical, unclassified, unknown
PA3741	2.0	0.0025	PA3741	Hypothetical, unclassified, unknown
PA3742	3.8	0.0000	rplS	50S ribosomal protein L19
PA3743	2.9	0.0000	trmD	tRNA (guanine-N1)-methyltransferase
PA3761	2.9	0.0000	PA3761	Transport of small molecules
PA3865.1	6.6	0.0000	PA3865.1	pyocin S4 immunity protein
PA3866	6.4	0.0000	PA3866	pyocin protein
PA3901	2.0	0.0000	fecA	Fe(III) dicitrate transport protein FecA
PA3967	2.2	0.0148	PA3967	Hypothetical, unclassified, unknown
PA3979	2.7	0.0000	PA3979	Hypothetical, unclassified, unknown
PA3990	3.6	0.0002	PA3990	Hypothetical, unclassified, unknown
PA4063	27.8	0.0000	PA4063	Hypothetical, unclassified, unknown
PA4064	8.4	0.0000	PA4064	Transport of small molecules
PA4065	9.5	0.0000	PA4065	Membrane proteins
PA4066	5.0	0.0000	PA4066	Hypothetical, unclassified, unknown
PA4153	2.0	0.0302	PA4153	"2,3-butanediol dehydrogenase"
PA4154	2.2	0.0008	PA4154	Hypothetical, unclassified, unknown

PA4272	2.0	0.0000	rplJ	50S ribosomal protein L10
PA4272.1	2.1	0.0000	PA4272.1	Non-coding RNA gene
PA4326	2.0	0.0007	PA4326	Hypothetical, unclassified, unknown
PA4385	2.2	0.0000	groEL	GroEL protein
PA4386	2.1	0.0000	groES	GroES protein
PA4420	2.3	0.0000	PA4420	Hypothetical, unclassified, unknown
PA4421	2.7	0.0000	PA4421	Hypothetical, unclassified, unknown
PA4520	2.4	0.0000	PA4520	Adaptation, Protection; Chemotaxis
PA4542	2.0	0.0000	clpB	ClpB protein
PA4565	2.0	0.0000	proB	glutamate 5-kinase
PA4566	2.5	0.0000	obg	GTP-binding protein Obg
PA4567	2.4	0.0000	rpmA	50S ribosomal protein L27
PA4568	2.2	0.0000	rplU	50S ribosomal protein L21
PA4582	2.1	0.0000	PA4582	Hypothetical, unclassified, unknown
PA4583	2.3	0.0000	PA4583	Hypothetical, unclassified, unknown
PA4629	2.5	0.0055	PA4629	Hypothetical, unclassified, unknown
PA4635	4.0	0.0000	PA4635	Hypothetical, unclassified, unknown
PA4746	2.0	0.0000	PA4746	Hypothetical, unclassified, unknown
PA4761	2.9	0.0000	dnaK	DnaK protein
PA4762	2.7	0.0000	grpE	heat shock protein GrpE
PA4763	17.1	0.0000	recN	DNA repair protein RecN
PA4825	3.3	0.0000	mgtA	"Mg(2+) transport ATPase, P-type 2"
PA4826	5.3	0.0000	PA4826	Hypothetical, unclassified, unknown
PA4834	4.1	0.0000	PA4834	Membrane proteins
PA4835	20.5	0.0000	PA4835	Hypothetical, unclassified, unknown; Membrane proteins
PA4836	57.5	0.0000	PA4836	Hypothetical, unclassified, unknown
PA4837	72.6	0.0000	PA4837	Membrane proteins; Transport of small molecules
PA4838	7.8	0.0000	PA4838	Hypothetical, unclassified, unknown
PA4888	3.1	0.0001	desB	Fatty acid and phospholipid metabolism
PA4889	2.9	0.0075	PA4889	Putative enzymes
PA4890	2.3	0.0007	desT	Transcriptional regulators
PA4892	2.3	0.0039	ureF	urease accessory protein UreF
PA4893	3.0	0.0000	ureG	urease accessory protein UreG
PA4918	2.9	0.0000	PA4918	Hypothetical, unclassified, unknown
PA4932	3.0	0.0000	rplI	50S ribosomal protein L9
PA4933	3.2	0.0000	PA4933	Membrane proteins
PA4934	3.8	0.0000	rpsR	30S ribosomal protein S18
PA4935	3.5	0.0000	rpsF	30S ribosomal protein S6
PA5049	2.4	0.0000	rpmE	50S ribosomal protein L31
PA5053	2.6	0.0000	hslV	heat shock protein HslV
PA5054	2.2	0.0000	hslU	heat shock protein HslU
PA5072	4.2	0.0000	PA5072	Adaptation, Protection; Chemotaxis
PA5082	2.5	0.0000	PA5082	Transport of small molecules

PA5084	2.1	0.0329	PA5084	Putative enzymes
PA5098	5.4	0.0412	hutH	histidine ammonia-lyase
PA5106	7.0	0.0129	PA5106	Hypothetical, unclassified, unknown
PA5217	2.2	0.0000	PA5217	Transport of small molecules
PA5315	2.8	0.0000	rpmG	50S ribosomal protein L33
PA5316	3.9	0.0000	rpmB	50S ribosomal protein L28
PA5401	3.7	0.0135	PA5401	Hypothetical, unclassified, unknown
PA5464	2.6	0.0000	PA5464	Hypothetical, unclassified, unknown
PA5466	2.9	0.0000	PA5466	Membrane proteins
PA5468	3.8	0.0000	PA5468	Transport of small molecules
PA5469	7.2	0.0000	PA5469	Membrane proteins
PA5470	5.5	0.0000	PA5470	Translation, post-translational modification, degradation
PA5471	5.5	0.0000	PA5471	Antibiotic resistance and susceptibility
PA5498	2.3	0.0000	PA5498	Motility & Attachment
PA5499	2.2	0.0000	np20	transcriptional regulator np20
PA5529	2.5	0.0000	PA5529	Membrane proteins; Transport of small molecules
PA5530	5.2	0.0000	PA5530	Membrane proteins; Transport of small molecules
PA5534	12.5	0.0000	PA5534	Hypothetical, unclassified, unknown
PA5535	14.2	0.0000	PA5535	Hypothetical, unclassified, unknown
PA5536	58.1	0.0000	PA5536	Transcriptional regulators; Adaptation, Protection
PA5537	3.4	0.0000	PA5537	Hypothetical, unclassified, unknown
PA5538	21.8	0.0000	amiA	N-acetylmuramoyl-L-alanine amidase
PA5539	16.4	0.0000	PA5539	Hypothetical, unclassified, unknown
PA5540	11.7	0.0000	PA5540	Hypothetical, unclassified, unknown
PA5541	5.4	0.0000	pyrQ	dihydroorotase

Table S2: Genes reduced at least 2 folds in PAO1 by cisplatin treatment.

Locus_Tag	fold change	P-Value	Gene name	Annnotation
PA0028	-2.4	0.0000	PA0028	Hypothetical, unclassified, unknown
PA0044	-2.3	0.0000	exoT	Secreted Factors (toxins, enzymes, alginate)
PA0391	-2.0	0.0000	PA0391	Hypothetical, unclassified, unknown
PA0744	-2.6	0.0000	PA0744	Putative enzymes
PA0745	-2.3	0.0000	PA0745	Putative enzymes
PA0746	-2.1	0.0000	PA0746	Putative enzymes
PA0747	-2.1	0.0000	PA0747	Putative enzymes
PA1245	-2.2	0.0000	PA1245	Membrane proteins; Secreted Factors (toxins, enzymes, alginate)
PA1246	-2.4	0.0000	aprD	alkaline protease secretion protein AprD
PA1247	-2.4	0.0000	aprE	alkaline protease secretion protein AprE
PA1248	-2.4	0.0000	aprF	Alkaline protease secretion outer membrane protein AprF precursor
PA1249	-2.3	0.0000	aprA	Secreted Factors (toxins, enzymes, alginate)
PA1317	-2.9	0.0000	cyoA	cytochrome o ubiquinol oxidase subunit II
PA1318	-2.9	0.0000	cyoB	cytochrome o ubiquinol oxidase subunit I
PA1319	-2.9	0.0000	cyoC	cytochrome o ubiquinol oxidase subunit III
PA1320	-2.7	0.0000	cyoD	cytochrome o ubiquinol oxidase subunit IV
PA1321	-2.8	0.0000	cyoE	cytochrome o ubiquinol oxidase protein CyoE
PA1698	-2.5	0.0000	popN	Type III secretion outer membrane protein PopN precursor
PA1699	-2.6	0.0200	PA1699	Protein secretion/export apparatus; Secreted Factors (toxins, enzymes, alginate)
PA1705	-3.0	0.0003	pcrG	regulator in type III secretion
PA1706	-3.0	0.0000	pcrV	type III secretion protein PcrV
PA1707	-3.7	0.0000	pcrH	regulatory protein PcrH
PA1708	-4.3	0.0000	popB	translocator protein PopB
PA1709	-4.4	0.0000	popD	Translocator outer membrane protein PopD precursor
PA1710	-2.6	0.0000	exsC	"ExsC, exoenzyme S synthesis protein C precursor."
PA1711	-2.6	0.0000	exsE	ExsE
PA1712	-2.7	0.0000	exsB	exoenzyme S synthesis protein B
PA1718	-2.5	0.0279	pscE	type III export protein PscE
PA1720	-2.2	0.0139	pscG	type III export protein PscF
PA1899	-2.2	0.0026	phzA2	Secreted Factors (toxins, enzymes, alginate)
PA1900	-2.1	0.0089	phzB2	Secreted Factors (toxins, enzymes, alginate)
PA1901	-3.0	0.0004	phzC2	Secreted Factors (toxins, enzymes, alginate)
PA1906	-2.0	0.0000	PA1906	Hypothetical, unclassified, unknown
PA2026	-2.8	0.0000	PA2026	Membrane proteins
PA2027	-90.0	0.0000	PA2027	Hypothetical, unclassified, unknown
PA2062	-2.6	0.0000	PA2062	Putative enzymes
PA2083	-2.1	0.0000	PA2083	Carbon compound catabolism
PA2191	-2.6	0.0000	exoY	Secreted Factors (toxins, enzymes, alginate)
PA2368	-2.1	0.0017	PA2368	Hypothetical, unclassified, unknown
PA2786	-2.4	0.0000	PA2786	Hypothetical, unclassified, unknown
PA3117	-4.6	0.0000	asd	aspartate semialdehyde dehydrogenase

PA3118	-8.8	0.0000	leuB	3-isopropylmalate dehydrogenase
PA3119	-47.6	0.0000	PA3119	Hypothetical, unclassified, unknown
PA3120	-40.2	0.0000	leuD	3-isopropylmalate dehydratase small subunit
PA3121	-51.8	0.0000	leuC	3-isopropylmalate dehydratase large subunit
PA3278	-3.0	0.0000	PA3278	Membrane proteins
PA3568	-3.1	0.0000	PA3568	Putative enzymes
PA3569	-3.5	0.0000	mmsB	3-hydroxyisobutyrate dehydrogenase
PA3570	-3.1	0.0000	mmsA	methylmalonate-semialdehyde dehydrogenase
PA3710	-2.7	0.0000	PA3710	Carbon compound catabolism
PA3792	-24.0	0.0000	leuA	2-isopropylmalate synthase
PA3841	-2.4	0.0000	exoS	Secreted Factors (toxins, enzymes, alginate)
PA3842	-3.3	0.0000	PA3842	Secreted Factors (toxins, enzymes, alginate); Chaperones & heat shock proteins; Protein secretion/export apparatus
PA3964	-8.9	0.0000	PA3964	Hypothetical, unclassified, unknown
PA3965	-10.4	0.0000	PA3965	Transcriptional regulators
PA4175	-2.1	0.0000	piv	Secreted Factors (toxins, enzymes, alginate)
PA4219	-2.1	0.0004	PA4219	Membrane proteins; Antibiotic resistance and susceptibility
PA4222	-2.4	0.0000	PA4222	Transport of small molecules
PA4223	-2.4	0.0000	PA4223	Membrane proteins; Transport of small molecules
PA4224	-2.3	0.0000	pchG	pyochelin biosynthetic protein PchG
PA4225	-2.2	0.0000	pchF	pyochelin synthetase
PA4226	-2.1	0.0000	pchE	dihydroaeruginoic acid synthetase
PA4229	-2.0	0.0000	pchC	pyochelin biosynthetic protein PchC
PA4694	-3.0	0.0000	ilvC	ketol-acid reductoisomerase
PA4695	-4.0	0.0000	ilvH	acetolactate synthase isozyme III small subunit
PA4696	-4.5	0.0000	ilvI	acetolactate synthase large subunit
PA5302	-2.5	0.0000	dadX	catabolic alanine racemase
PA5303	-2.6	0.0000	PA5303	Hypothetical, unclassified, unknown
PA5304	-2.5	0.0000	dadA	"D-amino acid dehydrogenase, small subunit"
PA5548	-2.8	0.0000	PA5548	Membrane proteins; Transport of small molecules
PA5549	-13.8	0.0000	glmS	glucosamine--fructose-6-phosphate aminotransferase
PA5550	-8.7	0.0000	glmR	GlmR transcriptional regulator

Table S3: Bacterial strains used in this study.

Strain	Relevant characteristics	Source or reference
P. aeruginosa		
PAO1	Prototypic non-mucoid wild-type strain	[1]
PAO1/Δ*recA*	*recA* knockout of PAO1 constructed by allelic exchange	[2]
PAO1/ Δ*recA* COM	*recA* knockout of PAO1 constructed by allelic exchange with *recA* complementation plasmid	[2]
PA14	Non-mucoid wild-type strain	[3]
CF clinical isolate 57388A	mucoid multiple-drug resistant (MDR) clinical isolate	[4]

Table S4: Primers used for the qRT-PCR analysis.

Reference gene		Target gene	
GAPDH_F	GACCGTTCCTCGACAAGTTC	exoS_F	GACGACGGCTATCTCTCCAC
GAPDH_R	TCAGCAGGCCTTCGAGATAG	exoS_R	GTTCGATATCCCGCTGACAT
gyrB_F	CACGTACGACTCTTCCAGCA	pscG_F	AGCACTGCCACGAAGAGG
gyrB_R	GAACACCATGTGGTGCAGAC	pscG_R	AGGCCAGGGCTTCCTGATAG
rpoD_F	GGGGCTGTCTCGAATACGTT	pscL_F	CGAGGTTTACCAGGAGCAGA
rpoD_R	GGGATACCTGACTTACGCGG	pscL_R	GTAGCGTTTCCTGGATCAGC

References:

1. Holloway, B.W. and A.F. Morgan, *Genome organization in Pseudomonas.* Annu Rev Microbiol, 1986. **40**: p. 79-105.
2. Miller, R.V. and T.A. Kokjohn, *Expression of the recA gene of Pseudomonas aeruginosa PAO is inducible by DNA-damaging agents.* J Bacteriol, 1988. **170**(5): p. 2385-7.
3. O'Toole, G.A. and R. Kolter, *Flagellar and twitching motility are necessary for Pseudomonas aeruginosa biofilm development.* Mol Microbiol, 1998. **30**(2): p. 295-304.
4. Hoffmann, N., et al., *Novel mouse model of chronic Pseudomonas aeruginosa lung infection mimicking cystic fibrosis.* Infect Immun, 2005. **73**(4): p. 2504-14.
